# Assessment of non-linearity in calorie–income relationship in Pakistan

**DOI:** 10.3389/fnut.2022.1025929

**Published:** 2022-12-14

**Authors:** Nadia Shabnam

**Affiliations:** Department of Health Professions Education, National University of Medical Sciences, Rawalpindi, Pakistan

**Keywords:** calorie–income, non-parametric regression, semi-parametric regression, single-index model, non-linear elasticities, Pakistan

## Abstract

This article considers the issue of assessing non-linearity in the relationship between calorie consumption and income using non-parametric and semi-parametric approaches. These methodologies are implemented on the cross-sectional household survey data conducted in Pakistan in 2010–2011. This framework takes account of the heterogeneity among families and potential non-linearity in the relationship. The findings show that the calorie–income elasticity is considerable and statistically significant across estimating methodologies. The results also demonstrate that the elasticity is larger for the substantially poorer households of the sample. By incorporating the explanatory variables in a manageable way in the parametric section of regression procedures, the semi-parametric analysis also reveals a slight increase in calorie response to increases in income at various income levels.

## Introduction

One of the most significant issues affecting the impoverished, in both developed and developing countries alike, is possibly inadequate nutrition. Malnutrition would make people less productive and make them more susceptible to illness, both of which would contribute to the continued poverty and further problems for the poor. Calorie consumption has been demonstrated to have a substantial correlation with both productivity and human health, making it one of the most significant aspects from the perspective of policymakers (1). On the one hand, the human body needs calories to preserve its natural metabolism. On the other hand, calorie consumption is the top priority for policymakers when creating programs helpful for the underprivileged parts of society. These policies, which are being implemented in various countries, can be categorized as (i) basic food subsidies, (ii) cash transfers, (iii) food vouchers, and (iv) conditional finance. The success of these policies is based on the strategy used in designing the program (2) or the sensitivity of food demand to changes in income (3). As a result, we decided to use calorie consumption as the subject of our research in this work. The role of income in calorie consumption continues to generate serious investigations, with contrasting results appearing throughout the literature. The debate regarding the size of the calorie–income relationship is well-documented in the literature [details are given in (4)]. Recently, Santeramo and Shabnam (5) well-summarized this debate by providing a meta-analysis of articles published on this issue in several countries of the world. Most of the studies in the literature used the parametric approach, while non-linear specifications were also in many studies. Following Gibson and Rozelle (6), only few studies used semi-parametric specifications to deal with the non-linearity of the calorie–income relationship (3, 7, 8).

Previous studies focusing on the parametric approach have revealed that the relationship between income and calorie is linear. While poleman (9) and Lipton (10) have argued that the calorie–income curve may be elbow-shaped for samples from the very poor category, indicating that share of food budget initially increases with the increase in income for the poor households. Similarly, Strauss and Thomas (11) reported that elasticity for the lowest decile increased up to 0.26 and then decreased to 0.03 for the highest decile. Thus, following Ravallion (12), the literature generally agrees that the calorie–income relationship is non-linear. It shows that with the increase in income, per capita calorie consumption increases and then tends to decrease with a further increase in income. However, non-linear specifications such as the quadratic term of income and expenditure may not always be appropriate to capture the non-linearity or shape of the calorie–income relationship.

Another way to capture this non-linearity existing in the calorie–income relationship is by using non-parametric procedures. Non-parametric smoothing techniques represent a set of flexible tools for analyzing unknown regression relationships. These techniques can search for appropriate non-linear forms that can best describe the available data and also provide useful tools for parametric non-linear modeling and helpful diagnostics.^1^ Gibson and Rozelle (6), Abdulai and Aubert (13, 14), Skoufias et al. (15), Babatunde et al. (1), Skoufias et al. (16), among others, used the non-parametric approach to capture the potential non-linearity in the calorie–income relationship. Although non-linear items in the calorie–income relationship can be investigated using a non-parametric technique in general, this approach is limited to bivariate relationships. When we take into account the impact of additional potential variables, the situation gets worse. The “curse of dimensionality” refers to the issues related to this non-parametric method. The precision of the non-parametric estimator diminishes as the component of X grows. Thus, some authors emphasize on this point and favor the use of parametric estimates to examine the impact of additional factors other than expenditure on the consumption of calories and nutrients. But in this study, we prefer to use semi-parametric regression methods to deal with the curse of dimensionality. This article aims at contributing to the body of knowledge regarding calorie–income estimation using current advancements in semi-parametric estimation methods and model selection as well (17) in order to address the non-linearity problem mentioned beforehand.

In general, semi-parametric methods combine parametric and fully non-parametric models in a specific mode. Semi-parametric methods are supposed to impose assumptions that are stronger than the fully non-parametric method but less restrictive than the parametric method of estimation. This allows the semi-parametric methods to trim down the effective dimension of the estimation problem, thus increasing the precision of estimation relative to that obtained by the non-parametric estimation, while allowing greater flexibility and lowering the risk of specification errors that are possible with the parametric model. Semi-parametric methods represent some widely accepted methods that provide a flexible estimation. However, the use of the semi-parametric approach is still very limited in the literature.

As a result, our goal in this article is to explore the calorie–income link by employing non-parametric and semi-parametric techniques for analyzing household survey data (2010–2011). In a fully non-parametric regression framework, we used the logarithm of per capita calorie intake conditional on the logarithm of per capita expenditure, while in a semi-parametric framework, some other control variables can be added. Here, we consider the partially linear regression approach and the semi-parametric single-index model from the family of semi-parametric specifications. Several potential options such as GAM specifications and parametric double-log specifications are available, and we must choose among them. We used a procedure proposed by Hasio et al. (18) to choose among these various competing parametric, non-parametric, and semi-parametric specifications.

Following the Introduction, in section “Methodology” of the article, we give an overview of both the non-parametric regression method and the semi-parametric regression approach. Data, models, and descriptive statistics are presented in section “Data.” Finally, in section “Results,” we present the estimated results and contrast them with the parametric results to draw conclusions about the study. Section “Discussion” concludes the study.

## Methodology

In this section, we provide an overview of the estimation techniques used to explore the issue of the calorie–income relationship.

### Non-parametric estimation method

In the non-parametric method, no assumption is made regarding the functional form of conditional mean function and assumed that *r*(*x*) satisfies the smoothness condition such as differentiability. The technical detail is given in Li and Racine (19). We use the local linear kernel regression to estimate *r*(*x*), and the procedure for this technique is given as follows. At any given point *x*, we run a weighted linear regression of Y on X. The weights are chosen for the observations of *Y*i** are higher for which *Xi* is close to *x* than the observations which are far from *x*. The estimate of *r*(*x*) is the predicted value from the local regression at *x*, and the estimated slope coefficient of local regression “$\hat{\beta }\left(x\right)$” is considered an estimate of the slope $\hat{r}\left(x\right)$. Let (*h*) be a sequence of positive numbers, known as the bandwidth that converges to 0 as *n* → ∞ .

### Semi-parametric estimation methods

Previous studies used two methods to incorporate other control variables in calorie demand models. For example, Subramanian and Deaton (20) split the sample according to household size and then estimated the non-parametric regression for the calorie–income relationship within each subsample. Strauss and Thomas (21) first used the non-parametric locally weighted smoothing scatter plot technique to capture the non-linear items in the calorie–income relationship and then used the log-inverse of the quadratic term (parametric functional form) to approximate the shape they observed in their non-parametric framework. The major advantage of using the parametric approach is that other potential control variables can be added to the model. In this article, we implemented new methods to incorporate the covariates into the non-parametric model that are semi-parametric methods, as follows: semi-parametric partially linear regression method (two or three studies implemented this methodology, as mentioned before) and semi-parametric single-index method (none of the studies in literature implemented this approach).

#### Partially linear model

The semi-parametric partially linear regression model combines both non-parametric and parametric components and is given as follows:


(2.1) $$Y_{j}=X_{j}^{\prime }\beta +G\left(R_{j}\right)+u_{j},j=1,\ldots ,n$$

where X_*j*_ is *q* × 1 vector, *β* is *q* × 1 vector of unknown parameters, *G* is an unknown function, and *R*_*j*_ ∈ ℤ^*p*^ . The finite-dimensional parameter *β* represents the parametric part, and the unknown function *G* (.) represents the non-parametric part of the model. The data are supposed to be independent and identically distributed random variables (i.i.d) that are given as follows:


(2.2) $$E\left(u_{j}|{\text{X}}_{j},R_{j}\right)=0$$

(2.3) $$E\left(u_{j}^{2}|{\text{X}}_{j}=x,R_{j}=r\right)=\sigma^{2}\left(x,r\right)$$

In the partially linear model, the foremost issue is the identification of *β* ; once this is carried out, an estimator of *G* (.) can be easily obtained. The partially linear model was first proposed by Robinson (22), and then Li and Racine (19) extended this work to handle the presence of qualitative variables in this model.

#### Single-index model

A semi-parametric single-index model has a form of a conditional mean function given as follows:


(2.4) $$Y=G\left(X^{\prime }\beta \right)$$

where *Y* is the dependent variable, *X* ∈ ℤ^*p*^ is the vector of covariates, *β* is an unknown parameter vector of order *p* × 1, and *G* is an unknown function. The quantity *X*′*β* is known as *single index* as it is scalar, even though *x* is vector. From Equation (2), we can see that our model is only a function of *X*′*β* because when the functional form of *G* (.) is unspecified, then the location parameter *α* cannot be identified. This implies that *Y* depends on *x* only by the way of linear combination of *X*′*β*, and the relationship is characterized by the link function *G* (.). Thus, the main statistical issue is to estimate *G* and *β* from the data (*Y*,*X*). Model (2) involves many widely used parametric models as special cases. Such as, if *G* is the identity function, then (2) is the linear model. If *G* is observed to be cumulative normal or logistic distribution, then Equation (2) is a discrete-choice logit or probit model. In a case where *G*is unspecified, Equation (2) gives a specification that is more flexible than a parametric model. Thus, the semi-parametric single-index model just like the partially linear model is designed to lessen the effects emerging due to the curse of dimensionality.

##### Identification condition

For the estimation of *β* and *G*, some restrictions are required for their identification. That is, *β* and *G* must be obtained through the population distribution of (*Y, X*), as follows:


(2.5) $$\text{E}\left[Y|x\right]=G\left(x^{\prime }\beta \right)$$

The identification conditions for the single-index model were first investigated by Ichimura (23), and then in the case of the binary response model, Manski (24) and Horowitz (25) presented identifiability conditions for the single-index model. The identification of *β* and *G* in a semi-parametric single-index model requires that

(a) *G*(.) cannot be a constant function; otherwise, *β* is not identified.

(b) Perfect multicollinearity is not permissible among components of *x*.

(c) *x* should include at least one continuous random variable. The intuition behind this can be explained by the following reason. Suppose *x* has only a binary (0–1 dummy) variable, then the range of *x* is finite as well as the range of *X*′*β* for any vector *β*. Of course, there exists an infinite number of functions *G*(.) and *β* vectors that satisfy the finite number of restrictions imposed by E[*Y*|*x*] = *G*(*x*′*β*). For more details, refer to the study by Horowitz (25), who explained this condition for a specific example.

(d) *x* should not include a constant term (intercept) as long as *β* does not include the location parameter. It should only be identifiable up to a scale. For example, E[Y|x]=G(x′β)andE[Y|x]=G(λ+θx′β)* are observationally equivalent models, where *λ* and *θ* are both arbitrary and not equal to zero and G* is defined by the relation G(λ+θω)*=Gω for all *ω* in the range of *X*′*β*. So, *β* and *G* cannot be identified, unless we imposed the restrictions that uniquely identify *λ* and *θ*. The restriction imposed on *λ* is called location normalization and involves that *X* should not include the intercept term. The restriction on *θ* is called scale normalization, and this can be attained by assuming the first component of *X* is equal to 1, that is, *β* has unit length (||*β*|| = 1), and this component is assumed to be continuous.

##### Ichimura’s method

Several estimation methods are available to estimate *β*, but we describe the estimation method proposed by Ichimura (23) and used this method for analysis. If the function *G* were specified, then Equation (2.5) would be a standard non-linear regression model and *β* could be estimated through a non-linear least square (NLS) method with possible weights by minimizing ${\sum_{i=1}^{n}\left[Y_{i}-G\left(X_{i}^{{}^{\prime }}\beta \right)\right]}^{2}$ with respect to *β*. Then, the estimator would be as follows:


(2.6) $$\hat{\beta }=\underset{\hat{\beta }\in Z^{a}}{\arg\min}\sum_{i=1}^{n}\eta \left(X_{i}\right){\left[Y_{i}-G\left(X_{i}^{{}^{\prime }}\beta \right)\right]}^{2}$$

However, if the function *G* is unknown, then we first need to estimate *G*(.). In this situation, the kernel method cannot be directly applied to estimate *G*(*X*′*β*) because both *β* and *β* are unknown. In this situation, we can estimate *Y*_*i*_ = *G*(*X*′*β*) + *ε*_*i*_ and E(*ε*_*i*_|*X*_*i*_) = 0 for a given value of *β* by using the kernel method, which is given as follows:


(2.7) $$G\left(X_{i}^{\prime }\beta \right)\equiv \text{E}\left[Y_{i}|X_{i}^{\prime }\beta \right]=\text{E}\left[g\left(X_{i}^{\prime }\beta \right)|X_{i}^{\prime }\beta \right]$$

If $\beta =\hat{\beta }G\left(X_{i}^{\prime }\beta \right)=g\left(X_{i}^{\prime }\beta \right)$, then $G\left(X_{i}^{\prime }\beta \right)\neq g\left(X_{i}^{\prime }\beta \right)$ if $\beta \neq \hat{\beta }$ in general. A leave-one-out non-parametric kernel estimator of $G\left(X_{i}^{\prime }\beta \right)$is given as follows:


(2.8) $${\hat{G}}_{-i}\left(X_{i}^{\prime }\beta \right)\equiv {\hat{\text{E}}}_{-i}\left(Y_{i}|X_{i}^{\prime }\beta \right)=\frac{{\left(nh\right)}^{-1}\sum_{j=1,j\neq i}^{n}Y_{j}\left(\frac{X_{j}^{,}-X_{i}^{\prime }\beta }{h}\right)}{{\hat{s}}_{-1}\left(X_{i}^{\prime }\beta \right)}$$

${\hat{s}}_{-i}\left(X_{i}^{\prime }\beta \right)={\left(nh\right)}^{-1}\sum_{j=1,j\neq 1}^{n}k\left(\frac{X_{j}^{\prime }-X_{i}^{\prime }\beta }{h}\right)$ . Thus, Ichimura (23) suggested the estimation of $G\left(X_{i}^{\prime }\beta \right)$ by replacing with the leave-one-out estimator ${\hat{G}}_{-i}\left(X_{i}^{\prime }\beta \right)$ and choosing *β* using the semi-parametric NLS method. In this method, Ichimura also used a trimming function to trim out the small values of ${\hat{s}}_{-i}\left(X_{i}^{\prime }\beta \right)$. Consider the following:


(2.9) $$A_{\upsilon }=\left\{s\left(x^{\prime }\beta \right)\geq \upsilon ,\forall \beta B\right\}$$

(2.10) $$A_{m}=\left\{x:\left|x-x^{{}^{*}}\right|\leq 2h\mathrm{for}\mathrm{some}x^{{}^{*}}\in A_{m}\right\}$$

υ > 0 is a constant, *B* is a compact subset in ℤ^*p*^, *A_ϑ_* ⊂ *A*_*m*_ as *n* → ∞, *h* → 0 than *A*_*m*_ get smaller too *A_ϑ_*. Thus, Ichimura (23) estimator is as follows:


(2.11) $${\hat{\beta }}_{I}=\arg\;\underset{\beta }{\text{min}}\sum_{i=1}^{n}{\left[Y_{i}-{\hat{G}}_{-i}\left(X^{\prime }\beta \right)\right]}^{2}\eta \left(x_{i}\right)1\left\{X_{i}\in A_{\vartheta }\right\}$$

*η*(*X*_*i*_) is a non-negative weight function that is bounded in *A_ϑ_*, I(.) is an indicator function, 1{*X*_*i*_ ∈ *A_ϑ_*} is a trimming function that equals 1 if *X*_*i*_ ∈ *A_ϑ_*, or zero otherwise. The trimming function provides guarantee that the random denominator in the kernel estimator is non-negative, with high probability so as to simplify the asymptotic analysis.

### Model specification test

The Hsiao test is based on the moments that hold value zero if a parametric specification (H_0_) is correct, or greater than zero otherwise. In this case, the null hypothesis is given as follows:


$$H_{0}^{a}=\text{E}\left(Y|x\right)=\theta \left(x,\beta_{0}\right)=1forsome\beta_{0}\in \text{B}\subset {\mathbb{Z}}^{p}$$

where *θ*(*x*, *β*_0_) is a known function with *β*_0_ as a vector of unknown parameters of order *p* × 1. Under the alternative hypothesis, we have a function that is negation of $H_{0}^{a}$ :


$$H_{1}^{a}=\text{E}\left(Y|x\right)=m\left(x\right)\neq \theta \left(x,\beta_{0}\right)<1forall\beta_{0}\in \text{B}$$

The test statistics are based on *I* = E{*U*E(*U*|*X*)*f*(*X*)}, where *U* = *Y* − *θ*(*x*, *β*_0_) is independently proposed by Fan and Gijbels (26) and Zheng (27). Consider that *I* = E{[E(*u*_*i*_|*x*_*i*_)]^2^*f*(*x*_*i*_)} ≥ 0 and *I* = 0 if the null hypothesis is true. Thereby, *I* is a valid candidate for testing $H_{0}^{a}$ . The sample analog of *I* is given as follows:


(2.12) $$I_{n}=\frac{1}{n}\sum_{i=1}^{n}{\hat{u}}_{i}{\hat{\text{E}}}_{-i}\left(u_{i}|x_{i}\right){\hat{f}}_{-i}\left(x_{i}\right)$$

$$=\frac{1}{n}\sum_{i=1}^{n}{\hat{u}}_{i}\left\{n^{-1}\sum_{j=1,j\neq i}^{n}{\hat{u}}_{j}K_{\omega ,ij}\right\}$$

$$=\frac{1}{n^{2}}\sum_{i=1}^{n}\sum_{j=1,j\neq i}^{n}{\hat{u}}_{i}{\hat{u}}_{j}K_{\omega ,ij}$$

where ${\hat{u}}_{i}=y_{i}-\theta \left(x_{i},\hat{\beta }\right)$ is the residual of the parametric null model, $\hat{\beta }$ is $\sqrt{n}$ consistent estimator of *β, K*_*ω,ij*_ = *M*_*h,ij*_*W*_*γ,ij*_(*ω* = (*h, γ*)), and ${\hat{\text{E}}}_{-i}\left(u_{i}|x_{i}\right){\hat{f}}_{-i}\left(x_{i}\right)$ is the leave-one-out kernel estimator of E(*y*_*i*_|*x*_*i*_)*f*(*x*_*i*_). A CV method is used for the selection of *h* and *γ*, and ${\hat{I}}_{n}$ is used to denote the CV-based test and can be defined the same way as *I*_*n*_ in previous equation but replacing them (*h*_1_…*h*_*q*_, *γ*_1_…*γ*_*r*_)with CV smoothing parameters$\left({\hat{h}}_{1}\ldots {\hat{h}}_{q},{\hat{\gamma }}_{1}\ldots {\hat{\gamma }}_{r}\right)$. The rejection region for the test at the *α* level of significance is *J*_*n*_ > *c*_*α*_, and the critical value *c*_*α*_ can be obtained by the wild bootstrap method. For detail about the wild bootstrap method, see the monograph of Li and Wang (28), Li and Racine (19, pp. 357), and Hsiao et al. (29).

## Data

This study uses data of Household Integrated and Economic Survey (HIES) 2010–2011 (30), carried out from July 2010 to June 2011. The sample comprises 16,341 households and is a nationally representative survey covering 14 large cities and 81 districts, as well as urban and rural areas. The HIES also reports information on a variety of social issues, including education, health, employment and income, immunization, use and satisfaction with facilities and services, and household consumption and details. In the consumption module, the survey collects information on the quantities and values of 69 food items, and along with this, the survey also records information on 79 non-food items. The food consumption module of the HIES provides the main data for our analysis.

To compute the calorie consumption amount from the reported food quantities, we applied the conversion factors from Food Composition Table for Pakistan (31), which contains data on nutrient contents for various food items [for details of data extraction, refer to the study by (4)]. For example, the nutrient consumed of a particular type like calorie is as follows:


$$N\Sigma \theta_{i}Q_{i}$$

where *N* is the quantity of the particular nutrient consumed, *θ*i** is the average nutrient content of a unit of food *i*, and *Q*i** is the number of units consumed of food *i* (4).

For the purpose of analysis, we have *Y* (the response variable) as logarithm of per capita daily calorie consumption, and control variables on the right-hand side are logarithm of per capita expenditure (ln_PCME), household size (HHsize), gender of household head (F_HHH), age of the household head (Age_HHH), and employment status of the household head (E_Status). There are also other potential variables that can be used, but we used the dimension reduction method named least absolute shrinkage and selection operator (LASSO)^2^ method to select the variables to better explain these estimation procedures. The flaw of the non-parametric method of considering only the bivariate relationship can be handled by using semi-parametric methods by including other covariates in the model in a tractable manner, but, in our study, explanatory variables were around 26, and data size was also large enough, so it is not feasible to include the entire set of variables in the semi-parametric method and obtain the results. Thus, we used LASSO as a variable selection procedure in order to ease the computational burden and increase the prediction accuracy.

Stepwise regression normally chooses models that include just a subset of the variables, while ridge regression includes all variables in the final model, and the penalty factor (θ) in ridge regression shrinks the coefficients toward zero but does not set any of the coefficients exactly to zero and does not exclude any of those from the final model (32). The LASSO overcomes this disadvantage of ridge regression. The LASSO minimizes the quantity as follows:


(3.1) $$\sum_{i=1}^{n}{\left(y_{i}-\beta_{0}-\sum_{j=1}^{q}\beta_{j}x_{ij}\right)}^{2}+\theta \sum_{j=1}^{q}\left|\beta_{j}\right|\;RSS+\theta \sum_{j=1}^{q}\left|\beta_{j}\right|$$

Statistically, the LASSO uses an *ℓ*_1_ norm and a coefficient vector associated with this norm is as ||*β*||_1_ = ∑|*β*_*j*_| . The LASSO not only shrinks the coefficients toward zero but also forces to be exactly equal to zero when parameter *θ* is sufficiently large. Consequently, the model generated can be easily interpreted and produced by ridge regression [for more details, see (32)]. We first used LASSO for the variable selection in the linear model in this study, then we used the same set of explanatory variables (excluding expenditure variable) for the parametric part in semi-parametric methods.

## Results

A sample of 16,290 households were used for the analysis. Descriptive statistics for the variable used in the study are given in Table 1. For non-parametric estimation, we restricted our analysis to only per capita expenditure and used it as the independent variable to avoid the curse of dimensionality. The results obtained from LASSO show that per capita expenditure first enters the model. Then, E_Status, shortly followed by F_HHH, HHsize, and Age_HHH, simultaneously enters in the model.

**TABLE 1:** Summary statistics of the variables used in semi-parametric models.

Variables	Mean	Standard deviation	Minimum	Maximum
ln_PCDCC	7.86	0.28	6.43	8.98
ln_PCME	7.94	0.53	6.21	11.64
HHsize	6.68	3.04	1.00	25.00
F_HHH	0.08	0.27	0.00	1.00
Age_HHH	46.20	13.23	45.00	75.00
E_Status	0.20	0.40	0.00	1.00

The models that we estimated are as follows:


(4.1) $$\begin{array}{l} E\left(Y|\ln\_PCME,regressors\right)=\\ \beta_{0}+\beta_{1}.\ln\_PCME+\beta_{2}.HHsize+\;\beta_{3}\text{.F\_HHH+}\\ \beta_{4}.Age\_HHH+\beta_{5}.E\_Status \end{array}$$

(4.2) $$\begin{array}{l} E\left(Y|\ln\_PCME,regressors\right)=\\ G(\beta_{1}.\ln\_PCME+\beta_{2}.HHsize+\beta_{3}.F\_HHH+\\ \beta_{4}.Age\_HHH+\beta_{5}.E\_Status) \end{array}$$

(4.3) $$\begin{array}{l} E\left(Y|\ln\_PCME,regressors\right)=\\ \beta_{1}.HHsize+\beta_{2}.F\_HHH+\beta_{3}.Age\_HHH+\\ \beta_{4}.E\_Status+m(\ln\_PCME) \end{array}$$

(4.4) E(Y|ln⁡_PCME)=r(ln⁡_PCME)

where *G, m*, and *r* are unknown functions, and *β*’s are unknown model parameters that may have different values in different models. Model (4.1) is a parametric model; model (4.2) and (4.3) are semi-parametric in nature, particularly model (4.2) is a single-index model and (4.3) is a partially linear model; and model (4.4) is fully non-parametric. For model (4.1), the parameters were estimated by using the standard OLS method. In the semi-parametric single index model, scale normalization was attained by setting *β*_1_ = 1 using the non-linear least square method of Ichimura (23). This method uses a kernel estimator to estimate the unspecified function *G*. In the case of partially linear regression model, *E*(*Y*|ln _*PCME*) and *E*(*regressors*|ln _*PCME*) were estimated by local linear regression using the second-order Gaussian kernel.


$$K\left(w\right)=\exp\left(-Z^{2}/2\right)/2\sqrt{\pi }$$

where *z* = (*x*_*i*_ − *x*)/*h* and *h* > 0. The fully non-parametric model (4.4) was estimated by using the local linear kernel method, and the method of least square cross-validation was used for the bandwidth selection in all estimation methods.

### Non-parametric results

**FIGURE 1:**
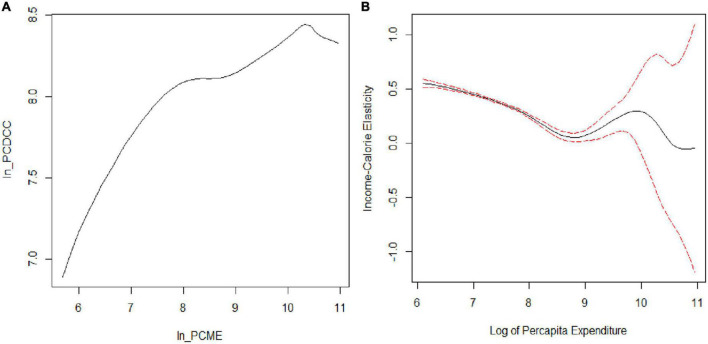
Non-parametric estimation of calorie–income relationship. **(A)** Calorie–expenditure curve and **(B)** income–calorie elasticity.

The calorie–expenditure curve in Figure 1A is positively sloped, then slightly flattens, and last demonstrates a sudden dip for very high-income group of expenditure distribution, but not flattens out at the tail. The possible reason for the dip could be the presence of outliers at the tail or the fact that the tail behavior in the non-parametric regression is not always good because of having few observations in the tails (33). Only 10% of the sample belongs to a very high-income group, and this may be the reason that the curve is not flattening out and showing a decreasing trend at the tail.

The gradients related to the non-parametric regression model provide a noticeable picture of the relationship. The gradients are shown in Figure 1B, which also shows 95% confidence bands for the gradients of the local linear non-parametric regression. Its shows the local linear fit by using a second-order Gaussian kernel method, with a CV bandwidth of 0.378 and bootstrapped standard error to construct a 95% confidence interval. The bootstrap procedure does not consider the cluster effect, thereby correcting the possible heteroscedasticity in errors. The procedure of bootstrapping was performed with 50, 100, and 200 replications, but in all procedures, the confidence bands obtained from standard errors were identical. Efron and Tibshirani (34) suggested that 200 replications are enough for the estimation of standard errors. In our case, the bands were fairly tight around the lower and middle of the regression and wide at the upper tail.

The income elasticity of calories with bootstrap standard error in Figure 1B shows that the curve slopes downward, which means calorie consumption falls less rapidly for poorer households because their income constraints either the quantity or quality of their food budget. The overall representation of this simple bivariate relation by using the non-parametric estimation method implies that calorie–income elasticity is statistically different from zero for almost all income levels, except for the very high-income level, where income elasticity is negative and insignificant, and it shows that local linear regression estimates the relationship with relative precision. We also ran a parametric regression of the log per capita daily calorie consumption on log of per capita expenditure to determine how well it demonstrates the true relationship by using the non-parametric model.

### Semi-parametric results

This section describes the results of semi-parametric regression methods. Table 2 shows the *β* parameter estimates of models (4.1–4.3). To get a clear picture as compared to the point estimate, we semi-parametrically modeled the relation between calorie consumption and expenditure for a given parametric specification of the effect of household characteristics on consumption of calories. The basic aim, throughout the analysis, is to explore the response of calorie consumption over a range of income distribution to income changes, rather than at a single point.

**TABLE 2:** Parameter estimates of parametric and semi-parametric methods.

Independent variables	Parametric model	Partially linear model	Single-index model
Constant	5.658*** (0.359)	–	–
ln_PCME	0.307*** (0.005)	–	1
HHsize	−0.007*** (0.001)	−0.005*** (0.001)	−0.010*** (0.002)
F_HHH	0.027*** (0.007)	0.033*** (0.006)	0.082*** (0.028)
Age_HHH	−0.001*** (0.000)	−0.002*** (0.000)	−0.002*** (0.001)
E_Stauts	0.175*** (0.004)	0.162** (0.004)	0.376*** (0.010)
R^2^	0.371	0.41	0.42

Standard errors are within parentheses. *, **, and *** indicate statistical significance at 10, 5, and 1%, respectively.

The income elasticity of calorie consumption is lower in multivariate parametric regression than in the bivariate regression model with the per capita expenditure as the only regressor (0.32). Indeed, there is a small difference between the parametric and partially linear estimates, but there is a relatively higher difference between parametric and single-index estimates.

**FIGURE 2:**
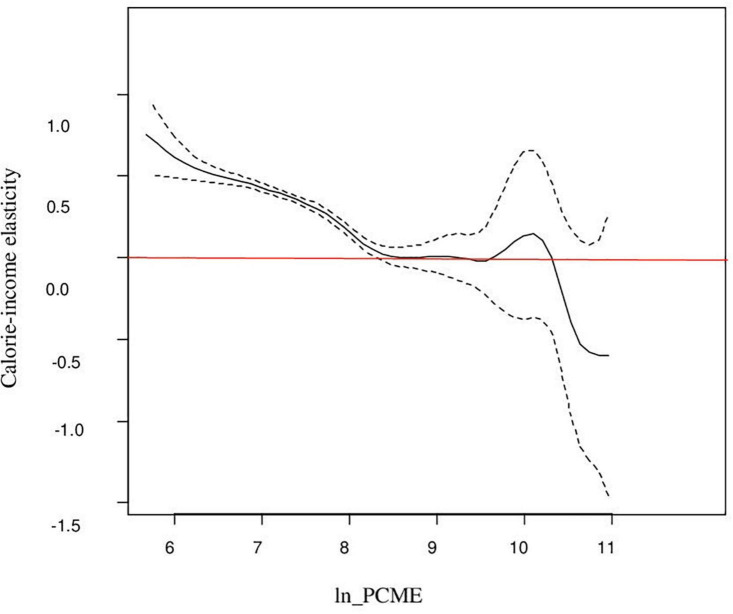
Semi-parametric partially linear estimation of calorie–income relationship.

Figure 2 shows the elasticity for different levels of income distribution and also demonstrates a higher and statistically significant estimate for the lower income group. The figure shows that expenditure elasticities are less than unity and remain fairly constant between 0.7 and 0.8 over a range of the low-income group. It is only at levels of Y above the sample mean of monthly per capita expenditure of Rs. 2,596 (in terms of log as 7.2). This elasticity decreases with the increase in income, and beyond the mean income, it begins to decrease and then becomes insignificant (as zero line is included in the confidence band). One possible reason for income elasticity being not statistically different from zero for the higher income group could be their interest in non-nutritive attributes of food items (33). Overall, the picture illustrates the fact that calorie consumption will improve with the change in income for poorer households as compared with their rich households. This result is consistent with the study of Subramanian and Deaton (20), Gibson and Rozelle (6), Tian and Yu (7), Nie and Sousa-Poza (8), and Trinh et al. (3). Trinh et al. (3) used semi-parametric specifications belonging to the family of generalized additive models to estimate the relationship for China and Vietnam. We also observed that the plot of non-parametric and semi-parametric regressions is almost the same in scale and shape, and this is consistent with the study of Bhalotra and Attfield (35) and Roy (33).

**FIGURE 3:**
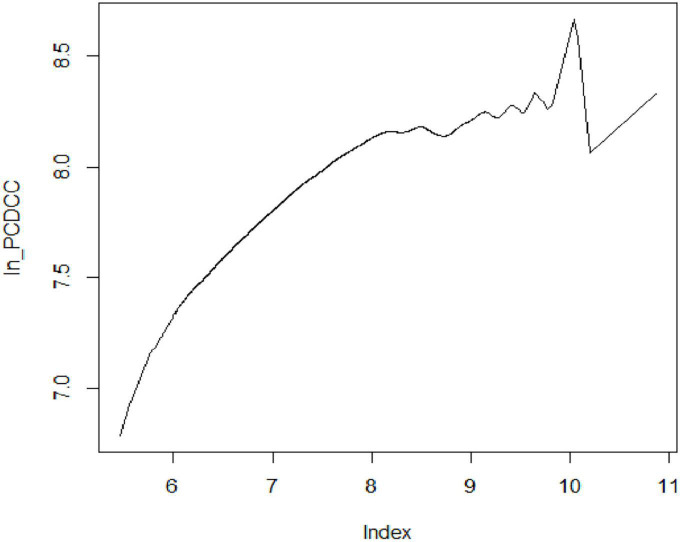
Semi-parametric single-index estimation of calorie–income relationship.

However, in terms of point estimate, the average elasticity is slightly higher in the semi-parametric partially linear regression model than in the fully non-parametric regression model. It shows that by adding covariates to the model causes only a small increase in the elasticity of calories. Moreover, Gibson and Rozelle (6) showed a slightly downward shift in elasticity estimates by adding covariates in the semi-parametric model. The coefficient of per capita expenditure in the single-index model is set by normalization. Thus, the eminent feature of the single-index model is that *E*(*Y*|*regressors*) is constant along curves such as ln _*PCME* + *β*_2_.*x*_2_ + *β*_3_.*x*_3_ + *β*_4_.*x*_4_ + *β*_5_.*x*_5_ is constant for the parameter *β* . The curve in Figure 3 shows that the index is increasing and has a similar trend as in the non-parametric model but has a fluctuating behavior at the upper end of the tail. However, providing an average value for non-parametric and semi-parametric models really wipes out the significant contribution of this kind of analysis.

The household size has a negative magnitude in all models (Table 2). It shows that economies of size decrease the calorie consumption by 0.5–1 in the percentage point. Similarly, age of the household head has a negative effect on household calorie consumption. Gender of the household head also has a positive and significant effect on calorie consumption. Results reported in Table 2 show that a female household head increases the calorie consumption by 3–8% compared with a male household head. In addition to this, if a household head is employed, then the head will perform better care of welfare of the members of household in terms of increasing calorie consumption. Finally, the last row in Table 2 provides the goodness of fit of the parametric and semi-parametric models. The value of R^2^ shows that the parametric fit is poor compared with the fit of semi-parametric models, and the single-index model has a better fit than the partially linear model. Thus, the single-index model emerges out to be a better specified semi-parametric model on the basis of goodness of fit.

We have also used some formal specification model tests (6.37–6.40) based on residual analysis for the purpose of comparison among models. Many procedures are available for testing a parametric model against its non-parametric alternative, but here, we used the test proposed by Hsiao et al. (29) due to its number of desirable properties in comparison to others. Hsiao et al. (29) proposed a non-parametric kernel-based model specification test and used a cross-validation (CV) method of bandwidth selection. This test used a residual-based wild bootstrap method to approximate the null distribution of the test statistics.

In our case, the implication of Hsiao’s test rejects the parametric model against the non-parametric model at the 1% level of significance (*J*_*n*_: 78.596, *p* < 0.001) and turns out to be significant for the non-parametric regression model. This formal specification test shows that the non-parametric model outperforms the usual parametric model, and this result is consistent with the results of the informal graphical analysis, as shown in Figure 1.

We also used Hsiao’s test to test the significance of semi-parametric methods with the parametric model in the null hypothesis, and again, this specification test rejects the parametric model (*J*_*n*_: 12.032, *p* < 0.001) and supports the implication of semi-parametric methods. Thus, the formal test is consistent with the informal graphical analysis, as shown in Figures 2, 3. This result is consistent with the findings of Trinh et al. (3), although they have used a preference test for model selection.

## Discussion

Non-parametric and semi-parametric estimation methods have attracted a great deal of attention from statisticians in the last decade. Horowitz and Lee (36) reported that the expediency of semi-parametric models in applied statistics is not well-understood in the literature yet, and any new application of semi-parametric models will generate valuable additional piece of information about these models. This article sheds light on the non-parametric and various semi-parametric estimators and demonstrated them with an application of consumption survey data (2010–2011) to identify the calorie–income relationship.

The analysis reported in this article shows that non-parametric and semi-parametric estimation methods achieved the proposed goal to capture the non-linearity in the calorie–income relationship. The fully non-parametric estimate embodies the true conditional mean function up to random sampling errors. Figure 1B shows a downward trend from the lower tail to the upper tail and demonstrates that calorie consumption decreases less rapidly for poorer households. Of course, the slopes at the extreme of the distribution are quite imprecisely estimated, but at the median level of expenditure, the slope is around 0.40 and is precisely estimated. However, it shows that local linear kernel regression estimates the relationship with relative precision. In addition to this, the article demonstrates the implication of two classes of semi-parametric regression: One is the partially linear model and the second is the single-index model. The plot of partial linear regression (Figure 2) shows that calorie consumption improves with the change in income for poorer households as compared with their rich counterparts. This result is consistent with the findings of Subramanian and Deaton (20) and Gibson and Rozelle (6), Tian and Yu (7), Nie and Sousa-Poza (8), and Trinh et al. (3). While the curve in Figure 3 shows that the index is increasing and has a similar trend as the non-parametric model but has a fluctuating behavior at the upper end of tail. Last, the comparison of non-parametric and semi-parametric estimation methods with the parametric method shows that the parametric fit is poor compared with the fit of the semi-parametric models, and the single-index model has a better fit than the partially linear model. Thus, the single-index model emerges to be a better specified semi-parametric model on the basis of goodness of fit. Moreover, the study revealed that fully non-parametric and semi-parametric models highlight the significant feature of the calorie–income relationship, which was not accounted for by using the parametric model.

## Strength and limitations

The calorie–income elasticity was calculated using information from a household survey. Otherwise, it would not have been possible for us to obtain comprehensive nutritional data from a large sample from different locations throughout Pakistan. In addition, this study concentrated on households in which the daily caloric intake ranged from 600 to 8,000 kcal. However, because of the sizeable sample size and thorough measurement of the overall calorie intake, this study was able to generate accurate estimations and significant insights into the general nutritional condition of the Pakistani population. From the methodological point of view, this study contributes to the literature the applying the single-index model and providing a test for model specification. The data used in the study are cross-sectional for a single year, but these methods can also be used for multiple waves of data from 2010 onward to get complete insights into the calorie–income relationship. We restricted the analysis to the calorie–income relationship, but the same methodology can also be applied to explore this relationship across different food groups, region-wise as well as gender-wise.

## Policy recommendations

The findings of this study suggest several significant policy changes that could be made to enhance the nutrition intake of the Pakistani population. The key concern is giving complete knowledge to eliminate nutritional gaps between average consumption and the ideal daily intake of calories in low-income households. This could be accomplished by increasing food subsidies, such as through networks of discounted grocery stores, direct nutrient supplementation plans, or in-kind transfers of food items, pricing interventions, cash transfer plans, and social safety net initiatives. Finally, an increase in money might not be enough to combat hunger; other socioeconomic and environmental issues, such as access to clean water, improved healthcare, and quality education, should also be taken into consideration. These elements might encourage better food consumption.

## Data availability statement

Publicly available datasets were analyzed in this study. These data can be found here: https://www.pbs.gov.pk/content/pakistan-social-and-living-standards-measurement.

## Author contributions

NS is the solo author of this manuscript, and conceptualized, analyzed, and interpreted the data and all the write-up.

## Footnotes

1 Details regarding non-parametric regression methods are given in Li and Racine (19).

2 Codes and results from LASSO can be provided on request.
